# Group 2 innate lymphoid cells drive inhibitory synapse formation with lasting effects on learning and memory

**DOI:** 10.1186/s12974-025-03485-5

**Published:** 2025-06-23

**Authors:** Johannes Steffen, Divija Deshpande, Henning Peter Düsedau, Janna Schmitz, Caio Andreeta Figueiredo, Laura Velleman, Claudia Pitzer, Christoph S.N. Klose, Ildiko R. Dunay

**Affiliations:** 1 Institute of Inflammation and Neurodegeneration, Otto-Von-Guericke University, Leipziger Straße 44, 39120 Magdeburg, Germany; 2 Department of Microbiology, Infectious Diseases and Immunology, Charité – Universitätsmedizin Berlin, corporate member of Freie Universität Berlin and Humboldt-Universität Zu Berlin, Berlin, Germany; 3 Department of Ophthalmology, Charité – Universitätsmedizin Berlin, Berlin, Germany; 4 Interdisciplinary Neurobehavioral Core, Ruprecht-Karls-Universität Heidelberg, Heidelberg, Germany; 5 Center for Behavioral Brain Sciences (CBBS), Magdeburg, Germany; 6 Center for Intervention and Research On Adaptive and Maladaptive Brain Circuits, Underlying Mental Health (C-I-R-C), Jena-Magdeburg-Halle, Magdeburg, Germany

## Abstract

The immune system provides multiple layers of protection that extend beyond conventional pathogen defense, including context-dependent modulation of behavior. However, the mechanisms driving these immune-mediated behavioral modifications remain incompletely understood. Here, we demonstrate that group 2 innate lymphoid cells (ILC2s) shape hippocampal synaptic development during early postnatal stages, with lasting effects on adult behavior, learning, and memory.

Using flow synaptometry, we identified a selective reduction in hippocampal VGAT^+^ GABAergic/glycinergic inhibitory synapse frequency at postnatal day 15 in ILC2-deficient mice, while the proportions of inhibitory GABAergic (NL2^+^) or excitatory glutamatergic (GluR1^+^) synapses remained unaltered. These synaptic changes occurred without detectable phenotypical changes in cortical and hippocampal microglia. In adulthood, ILC2-deficient mice displayed significant impairments in hippocampus-dependent tasks, such as active place avoidance and operant conditioning, reflecting deficits in learning and memory.

Our findings reveal a critical role for ILC2s in the formation of inhibitory synapses in the hippocampus, highlighting the impact of immune signaling on neuronal network maturation during a crucial period of brain development. This early immune-mediated modulation may have lasting effects on neuronal circuitry and cognitive functions that persist into adulthood, emphasizing the long-term implications of neuro-immune interactions for normal cognitive development and function.

**Supplementary Information:**

The online version contains supplementary material available at 10.1186/s12974-025-03485-5.

## Introduction

The immune system has been increasingly recognized for its effects on normal brain function, particularly through various meningeal subsets, such as T cells [1, 2], mast cells [3, 4], and innate lymphoid cells (ILCs) [5], which critically influence brain development, homeostasis, and behavior. During embryonic development and early life, innate immune responses are particularly influential, as innate immune cells seed the CNS and exert their functions during embryonic development before complementation by adaptive lymphocytes [6].

ILCs are a heterogeneous group of mainly tissue-resident innate immune cells that, aside their involvement in host defense, are also involved in the modulation of tissue development, homeostasis, repair, and remodeling. Although ILC subsets mirror the T cell subsets, they respond to local tissue cues rather than specific antigens. We and others have reported the compartmentalized distribution of different ILC subsets in the CNS and their role in inflammation, infection, and disease [7–11]. Group 2 ILCs (ILC2s) are the mediators of type 2 immunity, producing IL-4, IL-5, and IL-13 [12]. Classically associated with allergies and parasitic infections, type 2 immunity has recently emerged as a key regulator of CNS homeostasis and disease pathogenesis [13]. ILC2s begin to populate the dural meninges in newborn mice and retain this localization at the CNS border into adulthood [6]. Acute CNS trauma, such as spinal cord injury and intracerebral hemorrhage, results in activation of ILC2s and a subsequent accumulation at the site of injury [7, 14]. ILC2s contribute to restriction of neuroinflammation, limiting microglia and neutrophil activity via IL-13 signaling [15, 14] improving recovery after spinal cord injury [7] and reducing the lesion volume in case of intracerebral hemorrhage [14]. During aging, quiescent ILC2s accumulate in the choroid plexus and meninges. Their activation through IL-33 leads to the production of type 2 cytokines that repress aging-associated neuroinflammation and enhance cognitive function [16]. However, ILC2-specific roles in maintaining CNS homeostasis are still emerging, with many of their developmental and homeostatic functions under non-pathological conditions remaining elusive.

Our findings indicate that ILC2s promote inhibitory synapse formation in the hippocampus during early postnatal development, affecting locomotion and memory in adulthood. ILC2 deficiency resulted in hyperlocomotion and impaired hippocampus-dependent spatial learning and memory, while other behaviors (depression- and anxiety-like behaviors) remained unaffected. The lack of microglial alterations suggests that ILC2s may directly signal to neurons, bypassing traditional microglial-mediated synaptic pruning, to facilitate synaptic development. These findings highlight how innate immune signaling influences neuronal network maturation during postnatal brain development.

## Materials and methods

### Mice

*Nmur1*^*iCre−eGFP*^ [17, 18], *Id2*^fl/fl^ [19], and *Gata3*^fl/fl^ [20], on a C57BL/6 J background were bred under specific pathogen free (SPF) conditions. *Nmur1*^+/+^ *Id2*^fl/fl^ and *Nmur1*^+/+^ *Gata3*^fl/fl^ littermates served as controls for *Nmur1*^*iCre−eGFP/*+^ *Id2*^fl/fl^ and *Nmur1*^*iCre−eGFP/*+^ *Gata3*^fl/fl^ mice. Sex and age-matched male and female animals (15 or 30 days, or 7–18 weeks old) were used. Animals were group-housed on 12 h light / dark cycles at 22 °C with free access to food and water.

### Behavioral tests

All tests were performed in awake and unrestrained male mice during the day (light phase) in a double-blind fashion in mice aged 9–13 weeks. Age-matched littermate controls were included for each test.

#### Open field test

Anxiety/ exploratory activity of the mice was measured using the Open Field apparatus, which consisted of 40 × 40x40x cubicle [21]. The mice were placed in the cubicle and allowed to freely move for 10 min. The movement was tracked using a video camera mounted atop and analyzed using the software SYGNIS tracker v4.1.4. Analysis was based on a virtual grid dividing the arena into 25 squares of 64 cm^2^ each. More distance traversed or time spent on the edge of the area indicates anxiety-like behavior, whereas more activity in the centre squares indicates exploratory behavior.

#### Active Place Avoidance (APA)

The APA test apparatus [22] consisted of a rotating circular platform (1 rpm) located in a test cubicle. A randomly chosen 60° sector was designated as a non-rotating Shock Zone, wherein the mice received 0.4 mA electric current upon entry. Further identical shocks were would be received every 1.5 s if the mouse fails to leave the sector. For the mice, this sector was identifiable only using extra-maze visual cues. The shock was delivered using a cable, which could be attached to the metal clip inserted on the backs of the mice. Animal movement on the platform was recorded using a camera mounted on the top. The mice were placed on the platform for 10 runs (10 min each), each run conducted in intervals of 30 min. The first run was without shock followed by 8 runs with shock. The last run was performed without shock 24 h later. Over the period, the mice must learn to avoid the Shock Zone and remember the region 24 h later, thereby determining spatial learning and memory function of mice. Ethovision software was used to track the runs and analyze them.

#### Home cage monitoring

LABORAS home cage observation (Metris B.V.) system [23], consists of a carbon fiber platform to detect behavior-specific vibrations made by the mice. This system is used to detect changes is climbing, grooming, rearing, locomotion, immobilization, eating and drinking in absence of any stimuli under standard housing condition. The activity of the mice was measured for 24 h.

#### Tail suspension test

This test was used to analyze the depressive behavior of the mice [24]. In this test, the mouse is suspended upside down on a rod 60 cm above surface, by attaching an adhesive tape to its tail. The mouse activity was videotaped for 6 min and the latency to first immobilization and total immobilization duration was analyzed. A shorter latency or increased total immobilization period indicates depressive behavior.

#### Single chamber social interaction

Following 24 h of isolation, animals were habituated for 2 min in an open field arena (40 × 40 cm, 40 cm walls). Subsequently, same-sex littermate was introduced, interactions were recorded for 5 min, and analyzed using EthoVision XT (Noldus). Proximity was defined as ≤ 10 cm distance between the color markers on their backs.

#### Intellicage

Intellicage is a system used to measure different mouse behaviors (depending on the paradigm designed) in their natural state [25]. We used the TSE Systems setup [3]. and designed a paradigm for assessing spatial learning and memory function. Prior to test, the mice were subcutaneously implanted with a unique radiofrequency identification (RFID) chip into the nape under isofluorane anaesthesia. The Intellicage setup consisted of a large cage (housing upto 16 mice), with a water bottle placed in each corner. The bottle nozzle is enclosed in a chamber, which opens upon a nosepoke from the mouse. This chamber can be programmed to open only for a specific RFID signal is detected, which allows allotment of a specific corner water bottle access to each mouse. The paradigm we tested lasted for 9 days:

- Day 1–2 (adaptation)- free access to food and water.
- Day 3–6 (nosepoke)—nosepoke required to access water in all corners.
- Day 7 (place preference)—each mouse can access water only in one corner upon nosepoke.
- Day 8 (return)—return to home cage with free access to food and water.
- Day 9 (memory)—water access in all corner upon nosepoke.

Thus, the mice must first learn to access water upon nosepoke, then learn to access water from the corner designated to them and finally remember the corner in which water was accessible 24 h earlier. We tested 8–10 mice at a time using this paradigm and analyzed their activity (number of nosepokes, corner errors) using Intellicage Plus software.

### Tissue collection

Mice were killed and afterwards transcardially perfused with 60 mL ice-cold sterile PBS. Dural meninges were removed and stored in 10 mL digestion buffer (DMEM high glucose, 20 mM HEPES, 100 U/mL Penicillin, 100 µg/mL Streptomycin). Brains (with pia mater) were dissected, and the cortical and hippocampal regions were either stored in sterile ice-cold PBS for flow cytometry [11] or snap-frozen in liquid nitrogen for synaptosome or RNA isolation.

### Cell isolation

Cells were isolated as previously described for meninges [26] and brain [11] with slight alterations. Brains were minced, homogenized in dissection buffer (HBSS, 50 mM glucose, 13 mM HEPES, pH 7.3), filtered through a 70 μm cell strainer, and centrifuged (400 g, 20 min, 4 °C). The cells were separated via discontinuous, isotonic Percoll gradient (70% / 30%) centrifugation (800 g, 35 min, 4 °C, minimal acceleration / deceleration). Meninges were centrifuged (270 g, 10 min, RT), resuspended in 2 mL digestion buffer with 0.1 mg/mL DNAse I and 2.5 mg/mL Collagenase D, and incubated (30 min, 37 °C). Cells were separated by 10 passages through an 18G needle, digestion was stopped by 2 mL STOP solution (PBS w/o Ca^2+^ Mg^2+^, 2% (v/v) FBS, 10 mM EDTA, 10 mM HEPES), cells were filtered through 70 μm strainer, centrifuged (400 g, 5 min, 4 °C), resuspended in 1 mL digestion buffer, and centrifuged again (400 g, 5 min, 4 °C).

### Flow cytometry makers and staining, flow cytometer, analysis

Cells were resuspended in FACS buffer (PBS, 2% (v/v) FCS, 2 mM EDTA) supplemented with 1 µg anti-mouse CD16/32 antibody and a viability dye, and incubated (20 min, 4 °C). Surface staining was performed using fluorochrome- or biotin-conjugated antibodies against CD45, CD3, CD4, CD8, B220, CD11b, NK1.1, NKp46, CD25, TCRγδ, Ly6G, and Siglec F (30 min, 4 °C). Cells were washed (FACS buffer) and centrifuged (400 g, 10 min 4 °C) twice. If necessary, additional stained with fluorochrome-conjugated streptavidin was performed (30 min, 4 °C), followed by another washing / centrifugation step. For intracellular staining, cells were fixed and permeabilized with the Foxp3 / Transcription Factor Staining Buffer Set (60 min, 4 °C), washed / centrifuged twice, and stained using fluorochrome-conjugated antibodies against Eomes, T-bet, and GATA-3 (40 min, 4 °C). Cells were washed / centrifuged twice, resuspended in FACS buffer, acquired using an ID7000 Spectral Cell Analyzer (Sony Biotechnology) or Attune™ NxT flow cytometer (Thermo Fisher Scientific) with FMO controls assessing background fluorescence. Data were analyzed using FlowJo software (v10.8.2).

### Synaptosome isolation and flow synaptometry

Snap-frozen cortical and hippocampal tissues were used for crude synaptosome isolation via sucrose density centrifugation [27]. Synaptosomes were resuspended in SET buffer (320 mM sucrose, 1 mM EDTA, 5 mM Tris, pH 7.4) with 5% DMSO, aliquoted, slowly frozen (-1 °C / min), and stored (-80 °C) until further use [28]. Thawed synaptosomes were centrifuged (14,000 g*,* 10 min, 4 °C), fixed and permeabilized (Foxp3 / Transcription Factor Staining Buffer Set, 45 min, 4 °C), washed with SET buffer, centrifuged (14,000 g*,* 10 min, 4 °C), and resuspended in permeabilization buffer with 10% normal goat serum. Primary antibodies against synaptophysin (1:2000, #101308, Synaptic Systems), synaptobrevin 2 (1:1000, #104318, Synaptic Systems), GluR1 (1:400, #13185, Cell Signaling Technology), GluR1 (1:400, #ABN241, Merck Millipore), neuroligin 2-AlexaFluor™ 546 (1:200, #129511, Synaptic Systems), and VGAT-biotin (1:200, #131011BT, Synaptic Systems) were added and incubated (45 min, 4 °C), washed, and stained (45 min, 4 °C) with: goat anti-guinea pig-AlexaFluor™ 647 (1:200, #A21450), goat anti-rabbit-AlexaFluor™ 488 (1:4000, #A32731), streptavidin-BrilliantViolet 421™ (1:200, #405226, BioLegend). Synaptosomes were washed, stained with FM™4-64FX (0.2 µg/mL, #F34653), diluted, and acquired on Attune™ NxT flow cytometer (Thermo Fisher Scientific). Data were analyzed using FlowJo (v10.8.2).

### RNA isolation and RT-qPCR

Tissue samples were thawed in RNAlater, incubated overnight (4 °C), and homogenized in TRIzol™ [11]. RNA was isolated using peqGOLD total RNA kit (VWR International) and quantified NanoDrop 2000 Spectrophotometer.

Gene expression analysis was performed using TaqMan™ probes for *brain derived neurotrophic factor* (*Bdnf*), *synaptophysin* (*Syp*), *VGAT* (*Slc32a1*), *PSD-95* (*Dlg4*), *gephyrin* (*Gphn*), *Vglut1* (*Slc17a7*), *GABA A receptor, subunit alpha 1* (*Gabra1*), *Eaat2* (*Slc1a2*), *complement component 1, q subcomponent, alpha polypeptide* (*C1qa*), *complement component 3* (*C3*), *Rh-related antigen, integrin-associated signal transducer* (*Cd47*), *triggering receptor expressed on myeloid cells 2* (*Trem2*), *scavenger receptor class D, member 1* (*Cd68*), with the TaqMan™ RNA-to-CT™ 1-Step Kit on the LightCycler 96 (Roche Diagnostics) [11]. Mean Cq values were calculated using LightCycler 96 software (v1.1.0.1320), normalized to hypoxanthine guanine phosphoribosyl transferase (*Hprt*) expression using the 2^−ΔCq^ method (with ΔCq = Cq _Target gene_ − Cq _*Hprt*_) as previously described [29], and subsequently z-score transformed across all mouse lines, genotypes, and age-groups in Excel® 2019 (v16.0.10415.20025, Microsoft®).

### Quantification and statistical analysis

The statistical tests and sample sizes are stated in the figure legends. Normality (D’Agostino-Pearson for *n* ≥ 8, Shapiro–Wilk normality test for *n* < 8) and equality of variances (F test) were assessed in advance for selection of the statistical test. Sample sizes were based on literature and prior experience to ensure statistical power. Investigators were blinded during experiments and analysis when possible. Statistical calculations were performed in Prism (v10.2.3, GraphPad) and considered significant if *p* ≤ 0.05. Data are presented as arithmetic mean ± SEM, unless stated otherwise.

## Results

**Fig. 1:**
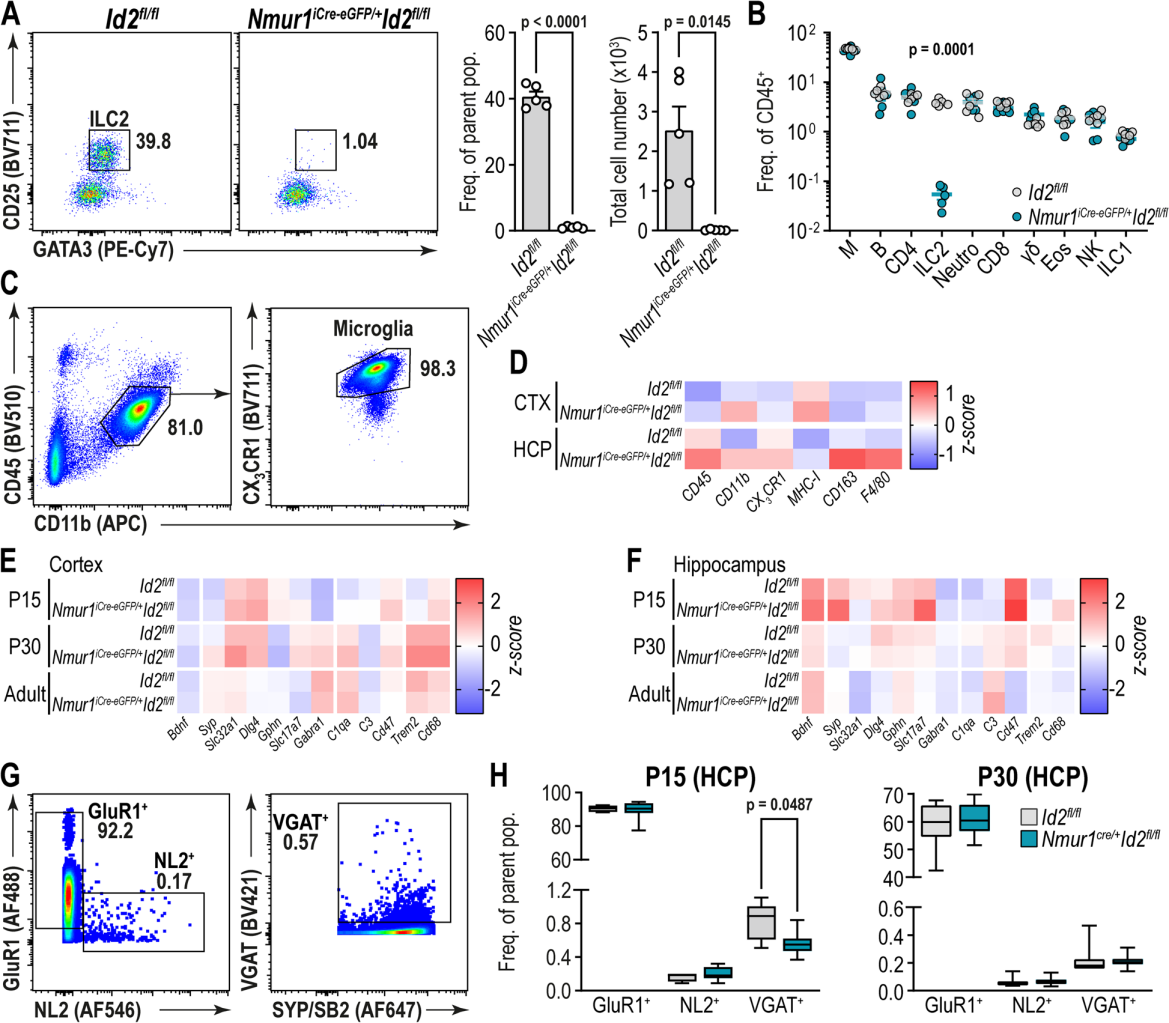
ILC2-deficiency impairs early postnatal development of hippocampal VGAT^+^ inhibitory synapses. **A** Flow cytometry plots of meningeal cells, pre-gated single live CD45^+^CD11b^−^CD33^−^B220^−^NK1.1^−^NKp46^−^ (not shown) to analyze Gata3^+^CD25^+^ ILC2s. Bar graphs (**B**) Flow cytometric analysis of meningeal cells, gated on single live CD45^+^ to identify myeloid cells (M: CD11b^+^Ly6G^−^SiglecF^−^), eosinophils (Eos: CD11b^+^Ly6G^−^SiglecF^+^), neutrophils (Neutro: CD11b^+^Ly6G^+^SiglecF^−^), T helper cells (CD4: CD11b^−^CD3^+^CD4^+^CD8^−^), cytotoxic T cells (CD8: CD11b^−^CD3^+^CD4^−^CD8^+^), γδ T cells (γδ; CD11b^−^CD3^+^CD4^−^CD8^−^TCRγ/δ^+^), B cells (B: CD11b^−^CD3^−^B220^+^), NK cells (NK: CD11b^−^CD3^−^B220^−^NKp46^+^NK1.1^+^Eomes^+^), ILC1s (ILC1: CD11b^−^CD3^−^B220^−^NKp46^+^NK1.1^+^Eomes^−^), and ILC2s (ILC2: CD11b^−^CD3^−^B220^−^NKp46^−^NK1.1^−^Gata3^+^CD25^+^). **C** Representative flow cytometry plots of cells isolated from the cerebral cortex, pre-gated on single live cells to identify CD11b^+^CD45^+^CX_3_CR37^+^ microglia. **D** Expression levels of indicated surface protein on CD11b^+^CD45^+^CX_3_CR37^+^ microglia in cerebral cortex (CTX) and hippocampus (HCP). **E**–**F** z-score standardized gene expression (normalized to reference gene) of indicated genes in cortex and hippocampus. **G** Representative dot plots show flow synaptometric analysis of crude synaptosomes from hippocampus. Isolated synaptosomes were first gated on size (300-1000 nm) and signal for FM™4-64FX, then for expression of synaptophysin and synaptobrevin 2 (Syp/SB2 + , not shown). Syp/SB2 + synaptosomes were further discriminated by expression of GluR1 (excitatory glutamatergic), neuroligin 2 (NL2, inhibitory GABAergic) or VGAT (inhibitory GABAergic/glycinergic). **H** Bar charts display the corresponding synaptosomal subtypes in HCP of ILC2-deficient mice and littermates as frequency of the parental population at P15 and P30. The plots (A, C, G) show concatenated samples (A, C: *n* = 5 mice; G: *n* = 14 mice). Data are presented as (D) Z-scores of respective MFI or (E) 2.^−ΔCq^ value. Analyses were performed at postnatal day 15 (E, F, G, H), 30 (E, F, H) or 8–18 weeks of age (A-F), and data was analyzed by (A) Welch’s t test, (B) multiple unpaired t tests with Welch correction (False Discovery Rate Q = 1.00%, two-stage step-up method of Benjamini, Krieger, and Yekutieli), (F) unpaired t test, (H) multiple unpaired t tests (Holm-Šídák)

To investigate the impact of ILC2s on brain function, we utilized *Nmur1*^*iCre−eGFP/*+^ *Id2*^*fl/fl*^ mice, which lack ILC2s in peripheral organs, such as the small intestine, mesenteric lymph nodes, and lung [17]. ILC2s are enriched in CNS border regions, particularly the meninges [7]. *Nmur1*^*iCre−eGFP/*+^ *Id2*^*fl/fl*^ specifically lacked GATA-3^+^ CD25^+^ ILC2 in the meninges (Fig. 1A), with unchanged frequencies of macrophages, B cells, CD4^+^, CD8^+^ and γδ T cells, neutrophils, eosinophils, NK cells and ILC1s (Fig. 1B).

Building on the evidence for meningeal immune regulation of parenchymal microglia and neurons [30], we assessed phenotypical markers in cortical and hippocampal microglia under steady-state conditions (Fig. 1C). No changes in the surface expression levels of CD45, CD11b, CX3CR1, MHC-I, CD163, and F4/80 were detected (Fig. 1D), suggesting normal microglial development. Gene expression levels were analyzed to screen for generalized alterations in immune response, neuronal function, and synaptic activity across developmental stages (Fig. 1E-F, Suppl. Figure 1A-B), revealing parallel changes in gene expression levels during development without significant alterations in ILC2-deficient animals. To investigate synaptic alterations, we performed flow synaptometric analyses at postnatal day (P)15 and P30 [27] to screen for synaptic changes during and after the critical postnatal development phase [31]. Quantification of excitatory glutamatergic (GluR1^+^), inhibitory GABAergic (NL2^+^), and inhibitory GABAergic/glycinergic (VGAT^+^) synapses (Fig. 1G), revealed a transient reduction of VGAT^+^ inhibitory synapses in the hippocampus at P15 (Fig. 1H), which normalized by P30. In contrast, no significant changes in frequencies of excitatory glutamatergic (GluR1^+^), inhibitory GABAergic (NL2^+^), or inhibitory GABAergic/glycinergic (VGAT^+^) synapses were detected in the cortex during this period (Suppl. Figure 1F). While genetic sex was not determined at P15, VGAT^+^ inhibitory GABAergic/glycinergic synapses showed sex-specific differences in the cortex but not the hippocampus at P30 (Suppl. Figure 1G-H). Given that altered hippocampal GABA levels can affect behaviors, such as general activity, anxiety-like behaviors, and social interaction [32], we hypothesized that the decreased number of inhibitory synapses in ILC2-deficient *Nmur1*^*iCre−eGFP/*+^ *Id2*^*fl/fl*^ animals might lead to an imbalance in excitatory/inhibitory (E/I) signaling, potentially increasing neuronal excitability and affecting cognition, memory, or behavior.

In the CNS, immune-derived cytokines also function as regulators of social behavior, learning, and memory [33], we proceeded to characterize whether our mouse model could be used to assess the effect of ILC2s on brain neurons. Therefore, we conducted several behavioral tests, each one to assess a different brain function, using ILC2-proficient and ILC2-deficient mice.

**Fig. 2:**
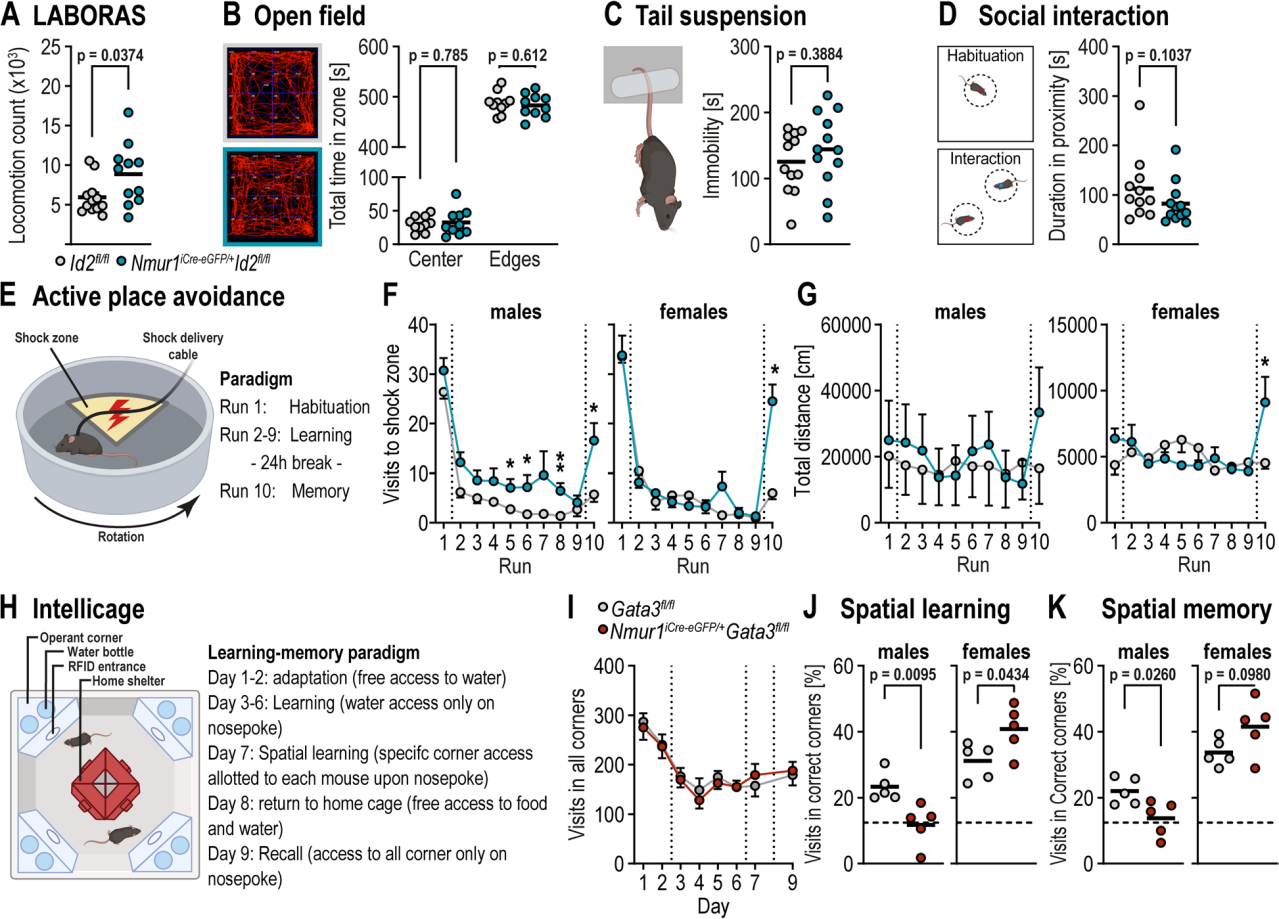
ILC2s promote hippocampus-dependent learning and memory. **A** Total locomotion counts of *Id2*^*fl/fl*^ and *Nmur1*^*iCre−eGFP/*+^ *Id2*^*fl/fl*^* mice*. **B** Open-field test (10 min) results, showing representative paths and time spend in the center or near walls of *Id2*^*fl/fl*^ and *Nmur1*^*iCre−eGFP/*+^ *Id2*^*fl/f*^.^l^ mice. **C** Total time of immobility during tail suspension test (6 min). **D** Duration of proximity (≥ 10 cm) during social interaction test (5 min). **E**  Active place avoidance test paradigm. **F** Total visits in shock zone for each run and (**G**) total distance for each run by sex. **H** Behavioral test paradigm for Intellicage. **I** Total number of visits in all corners (**J**, **K**) percentage of nose pokes in correct corner. Data was analyzed by (A, D) Mann–Whitney test, (B) multiple unpaired t tests with Welch correction (False Discovery Rate Q = 1.00%, two-stage step-up method of Benjamini, Krieger, and Yekutieli), (C, K) unpaired t test, (F, G, I) multiple unpaired t tests (* *p* < 0.05; ** *p* < 0.01), (J) Two way repeated measures ANOVA with Geisser-Greenhouse correction followed multiple comparison test (Holm-Šídák)

First, tested the mice for any general deviations in their daily activities using the home cage monitoring system (LABORAS). Upon observing all their activities for 24 h, we noticed a significant increase in locomotion duration of the *Nmur1*^*iCre−eGFP/*+^ *Id2*^*fl/fl*^ mice compared to their wild-type littermates (Fig. 2A). This prompted us to investigate into more specific behavioral modalities, which may underlie the changes in routine pattern observed in LABORAS. We therefore tested for the mice for exploratory/anxiety behavior using Open Field test (Fig. 2B) and for depressive behavior using the Tail Suspension test (Fig. 2C). We did not observe any significant changes in *Nmur1*^*iCre−eGFP/*+^ *Id2*^*fl/fl*^ mice in these tests. Although there was a tendency towards less social interactions when placed in a single chamber, this difference did not reach statistical significance for the cohort size tested (Fig. 2D, Suppl. Figure 2B). Finally, we explored spatial learning and memory function using the Active Place Avoidance (APA) test. In this test, a mouse is placed on a rotating turntable with a non-rotating region, which serves as ‘shock zone’ (Fig. 2E). The shock in this sector is mild (0.4 mA), enough to elicit aversion, but not fear. Over the course of 8 runs (10 min each), the mouse learns to avoid the shock zone using extra-arena visual cues and also remembers this region 24 h later (Fig. 2E). Interestingly, the *Nmur1*^*iCre−eGFP/*+^ *Id2*^*fl/fl*^ mice showed a significantly slower learning and retention compared to their wild-type littermates (Fig. 2B, C). This stark loss of spatial learning and memory was not attributed to significantly higher locomotive activity during the test (Suppl. Figure 2F). It must be noted, as opposed to LABORAS, APA test involves a stimulus. Hence, the differences in locomotive activities observed in both tests are reasonable. Importantly, the learning-memory deficit was pronounced in the males of the *Nmur1*^*iCre−eGFP/*+^ *Id2*^*fl/fl*^ mice. To validate the influence of ILC2s on behaviour alterations, we investigated similar behavioural modilities in *Nmur1*^*iCre−eGFP/*+^ *Gata3*^fl/fl^ mice. These mice exhibited the same phenotype as the *Nmur1*^*iCre−eGFP/*+^ *Id2*^*fl/fl*^ mice i.e. higher locomotive activity in LABORAS test (Suppl. Figure 3F) and impaired learning and memory as measured using Intellicage setup. Again, the learning-memory deficit was prominent in the males of *Nmur1*^*iCre−eGFP*^ *Gata3*^fl/fl^ mice (Fig. 2K, L), but no significant differences in Open field test (Suppl. Figure 3G), Tail suspension test (Suppl. Figure 3H) or social interaction (Suppl. Figure 3I).

Thus, loss of ILC2s is associated with a loss of cognitive function, specifically spatial learning and memory. With CNS ILC2s as known producers of the type 2 cytokines, these results are in line with [34] and [35]. We show for the first time that *Nmur1*^*iCre−eGFP/*+^ *Id2*^*flox/flox*^ and *Nmur1*^*iCre−eGFP/*+^ Gata^3flox/flox^ are reliable models to study ILC2-mediated behavioral patterns.

## Discussion

The role of the immune system in the development, homeostasis and function of the CNS has been increasingly recognized in recent decades [36]. However, the impact of ILCs remains less understood, partially due to their low abundance in the healthy CNS. Our research demonstrates that ILC2s are crucial for promoting hippocampal inhibitory synapse formation during postnatal development (Fig. 1G), aligning with recent findings that genetic ILC2 ablation delays inhibitory synapse formation in the cortex [37]. We also demonstrate that ILC2s support spatial learning and memory in young animals (Fig. 2E-K), building on prior studies showing ILC2 activation enhances memory in aged animals [16].

While the exact mechanisms require further exploration, ILC2-derived cytokines, such as IL-4 and IL-13, are known to influence learning and memory in adult animals [34, 38–40]. Structurally and functionally related IL-4 and IL-13 signal through heteromeric cell surface receptor complexes. IL-4 utilizes both, the type I complex (IL-4Rα / common γ chain heterodimer) and the type II complex (IL-4Rα / IL-13Rα1), whereas IL-13 only binds to the type II receptor complex [41]. A recent study reports a predominantly postsynaptic localization of IL-13Rα1, inducing rapid phosphorylation of NMDA and AMPA-type glutamate receptors in neurons, enhancing surface trafficking and synaptic activity [42], suggesting that IL-13 can affect learning and memory via modulation of synaptic plasticity. Under homeostatic conditions, the IL-4Rα subunit is expressed excitatory and inhibitory neurons, modulating the excitatory and inhibitory miniature postsynaptic potentials (mEPSCs and mIPSCs) [40, 34]. IL-4 increases the frequency of mIPSCs and decreases the frequency of mEPSCs [40]. Thus, neuronal IL-4Rα deficiency reduced the inhibition of excitatory neurons, resulting in increased exploratory behavior and locomotion, decreased anxiety levels, and impairments in fear learning [40].

Emerging evidence suggests that ILC2 exhibit sex-specific functional differences driven by immune and hormonal interactions. During allergic airway inflammation testosterone was shown to suppress proliferation and cytokine production of ILC2s [43]. In experimental autoimmune encephalomyelitis, males produce higher IL-33 levels, promoting a non-pathogenic Th2 response, whereas females, with a lower IL-33 response, develop a more pathogenic Th17-dominant response [44]. These findings suggest that sex influences the capacity of ILC2s to regulate CNS function, particularly in neuroinflammatory contexts. ILC2-derived cytokines also support cognitive functions via other cell types: IL-13 stimulates astrocytic BDNF production [39], and IL-4 promotes an anti-inflammatory phenotype in meningeal macrophages [35]. Beyond their physiological roles, ILC2s or their cytokines have been implicated in other CNS conditions, such as neurodegeneration [45], experimental autoimmune encephalitis (EAE) [44, 46], hypoxic-ischemic brain damage [47, 48], intracerebral hemorrhage [14], and traumatic brain injury [42, 49]. Thus, understanding and harnessing their regulatory functions may enable strategies to mitigate neurodegenerative diseases and facilitate recovery after CNS trauma.

In summary, meningeal ILC2s support CNS function through diverse pathways, including the promotion of inhibitory synapse maturation and modulation of learning and memory, emphasizing their potential as therapeutic targets.

### Limitations of the study

The synaptic analyses are based on flow synaptometry, which provides broad quantification of synapses, but lacks spatial resolution and direct functional assessment of synaptic transmission. While flow synaptometric results indicate that ILC2-mediated changes mainly affected inhibitory synapses, the specific neuronal subtypes of affected remain to be characterized.

## Supplementary Information

Supplementary Material 1: Figure 1: Genetic-ablation of meningeal ILC2s. (A) Representative dot plots show flow synaptometric analysis of crude synaptosomes from cerebral cortex. Isolated synaptosomes were first gated on size (300-1000nm) and signal for FM™4-64FX, then for expression of synaptophysin and synaptobrevin 2 (Syp/SB2+, not shown). Syp/SB2+ synaptosomes were further discriminated by expression of GluR1 (excitatory glutamatergic), neuroligin 2 (NL2, inhibitory GABAergic) or VGAT (inhibitory GABAergic/glycinergic). (B) Bar charts display the corresponding synaptosomal subtypes in CTX of ILC2-deficient mice and littermates as frequency of the parental population at P15 and P30. (C, D) Synaptosomal subtypes in HCP or CTX as frequency of the parental population at P30 from Fig. 1H or from D by sex. The plots (A) show concatenated samples (*n*=14 mice). Analyses were performed postnatal day 15 (A, B), or 30 (B-D) by (B, C, D) multiple unpaired t tests (Holm-Šídák).

Supplementary Material 2: Figure 2: Sex-specific analysis of behavioral effects. Results from (A) Open-field test (10 min), (B) social interaction test, (C), tail suspension test (6 min), and (D) Locomotion counts and immobility time of *Id2*^*fl/fl*^ and *Nmur1*^*iCreeGFP/+*^* Id2*^*fl/fl*^ mice from Fig. 2A-D by sex. Data was analyzed by (A) multiple unpaired t tests with Welch correction (False Discovery Rate Q=1.00%, two-stage step-up method of Benjamini, Krieger, and Yekutieli), (B, C, D-females) unpaired t test, (D-males) Welch’s t test.

Supplementary Material 3: Figure 3: Valifation in additional knock-out model. (A) Flow cytometry plots of meningeal cells, pre-gated single live CD45^+^CD11b^-^CD3^-^B220^-^NK1.1^-^NKp46^-^ (not shown) to analyze Gata3^+^CD25^+^ ILC2s. Bar graphs show comparison of frequency and total cell number. (B) Representative flow cytometry plots of cells isolated from the cerebral cortex, pre-gated on single live cells to identify CD11b^+^CD45^+^CX_3_CR37^+^ microglia. (C) Expression levels of indicated surface protein on CD11b^+^CD45^+^CX_3_CR37^+^ microglia in cerebral cortex (CTX) and hippocampus (HCP). (D, E) z-score standardized gene expression (normalized to reference gene) of indicated genes in cortex and hippocampus. (F)  Total locomotion duration of *Gata3*^*fl/fl*^ and *Nmur1*^*iCre-eGFP/+*^* Gata3*^*fl/fl*^* mice*. (G) Open-field test (10 min) results, showing time spend in the center or near walls of *Gata3*^*fl/fl*^ and *Nmur1*^*iCre-eGFP/+*^* Gata3*^*fl/f*l^ mice. (H) Total time of immobility during tail suspension test (6 min). (I) Duration of proximity (≥ 10 cm) during social interaction test (5 min). (J) Total number of visits in all corners and nose pokes by sex. The plots (A, B) show concatenated samples (*n*=4 mice), and (C) data are presented as Z-scores of respective MFI. The plots (A, B) show concatenated samples (A, B: *n*=4 mice). Analyses were performed at postnatal day 15 (D, E), 30 (D, E) or 8-18 weeks of age (A-J) by (A, I-females) Welch’s t test, (F, H-males) unpaired t test, (G) multiple unpaired t tests with Welch correction (False Discovery Rate Q=1.00%, two-stage step-up method of Benjamini, Krieger, and Yekutieli), (H-females, I-males) Mann-Whitney test.

## Data Availability

No datasets were generated or analysed during the current study.
