# Supplementary figures and images for: Group 2 innate lymphoid cells drive inhibitory synapse formation with lasting effects on learning and memory
Source: J Neuroinflammation. 2025 Jun 23;22:163. doi: 10.1186/s12974-025-03485-5 (PMC12183876; doi:10.1186/s12974-025-03485-5)

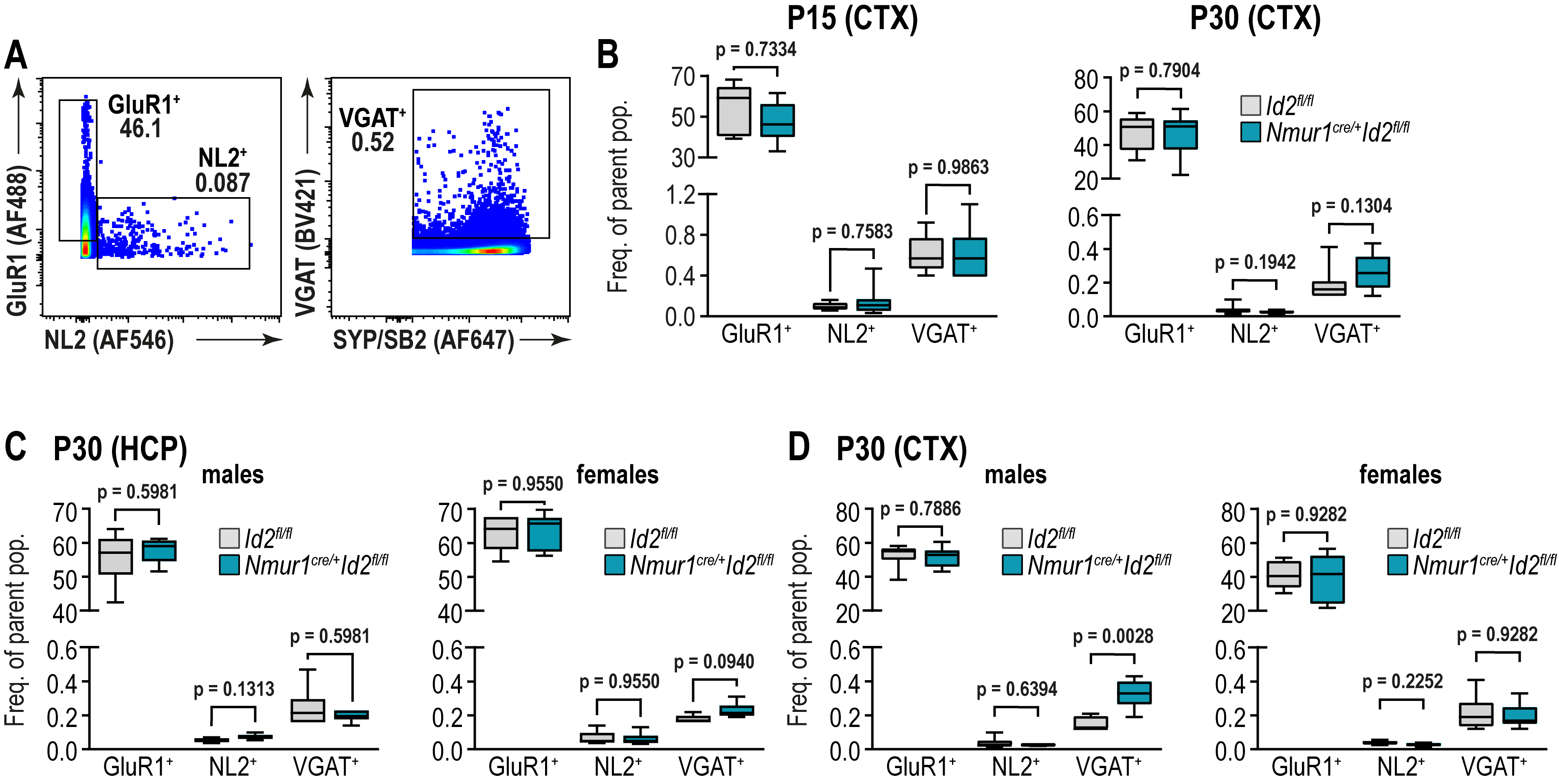

Supplement: Supplementary file 1 — Supplementary Material 1: Figure 1: Genetic-ablation of meningeal ILC2s. (A) Representative dot plots show flow synaptometric analysis of crude synaptosomes from cerebral cortex. Isolated synaptosomes were first gated on size (300-1000nm) and signal for FM™4-64FX, then for expression of synaptophysin and synaptobrevin 2 (Syp/SB2+, not shown). Syp/SB2+ synaptosomes were further discriminated by expression of GluR1 (excitatory glutamatergic), neuroligin 2 (NL2, inhibitory GABAergic) or VGAT (inhibitory GABAergic/glycinergic). (B) Bar charts display the corresponding synaptosomal subtypes in CTX of ILC2-deficient mice and littermates as frequency of the parental population at P15 and P30. (C, D) Synaptosomal subtypes in HCP or CTX as frequency of the parental population at P30 from Fig. 1H or from D by sex. The plots (A) show concatenated samples (n=14 mice). Analyses were performed postnatal day 15 (A, B), or 30 (B-D) by (B, C, D) multiple unpaired t tests (Holm-Šídák). [file 12974_2025_3485_MOESM1_ESM.png]

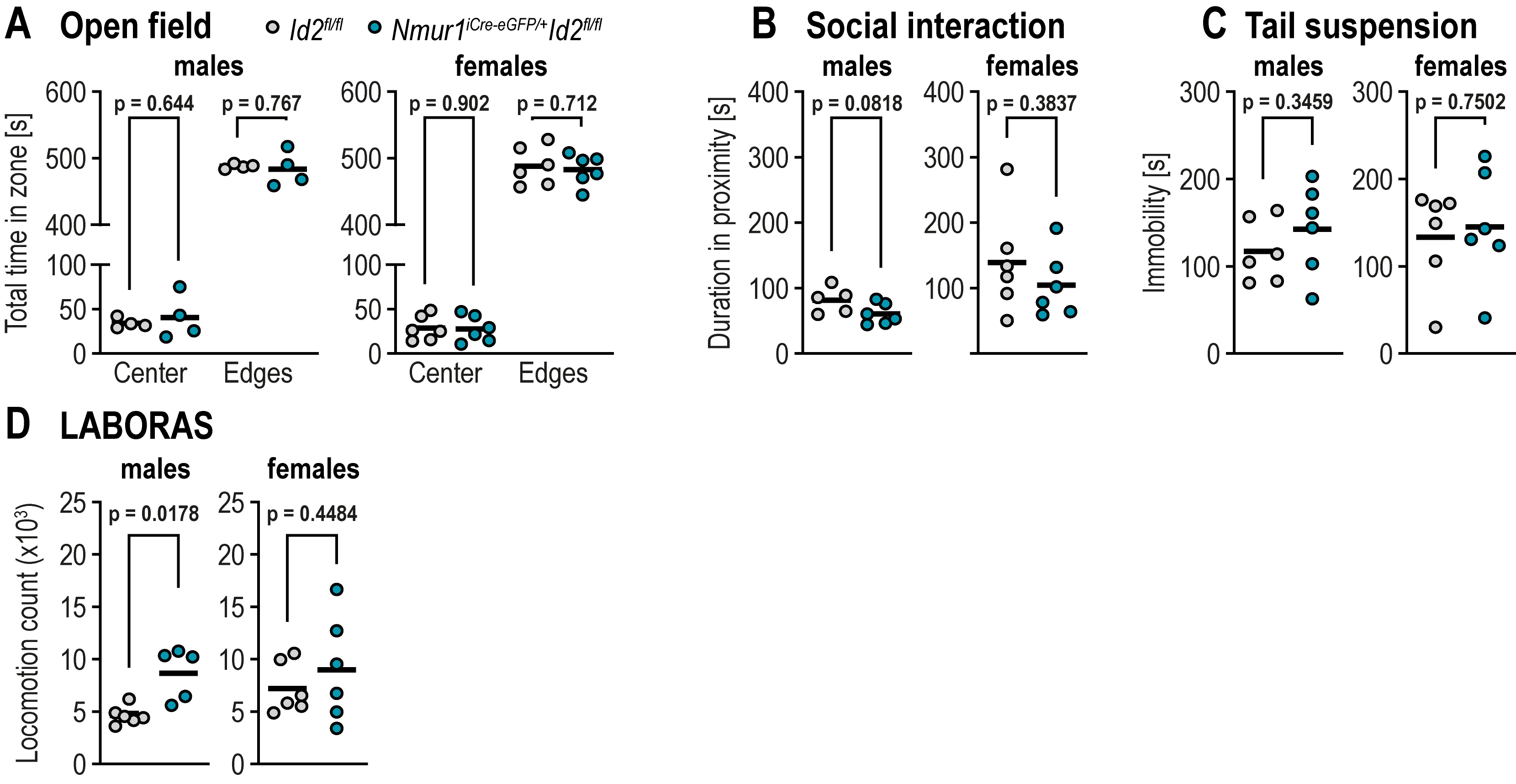

Supplement: Supplementary file 2 — Supplementary Material 2: Figure 2: Sex-specific analysis of behavioral effects. Results from (A) Open-field test (10 min), (B) social interaction test, (C), tail suspension test (6 min), and (D) Locomotion counts and immobility time of Id2fl/fl and Nmur1iCreeGFP/+ Id2fl/fl mice from Fig. 2A-D by sex. Data was analyzed by (A) multiple unpaired t tests with Welch correction (False Discovery Rate Q=1.00%, two-stage step-up method of Benjamini, Krieger, and Yekutieli), (B, C, D-females) unpaired t test, (D-males) Welch’s t test. [file 12974_2025_3485_MOESM2_ESM.png]

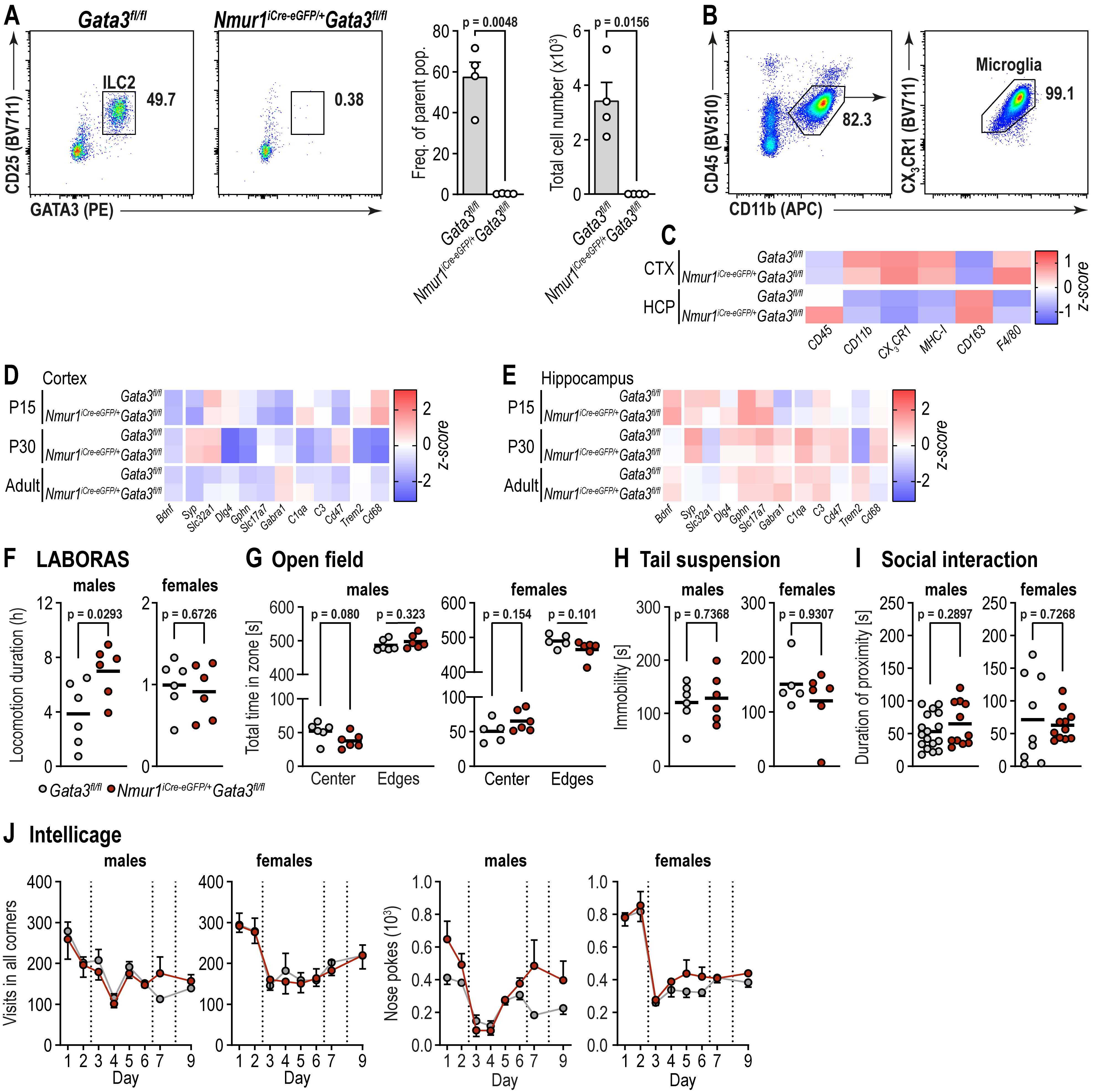

Supplement: Supplementary file 3 — Supplementary Material 3: Figure 3: Valifation in additional knock-out model. (A) Flow cytometry plots of meningeal cells, pre-gated single live CD45+CD11b-CD3-B220-NK1.1-NKp46- (not shown) to analyze Gata3+CD25+ ILC2s. Bar graphs show comparison of frequency and total cell number. (B) Representative flow cytometry plots of cells isolated from the cerebral cortex, pre-gated on single live cells to identify CD11b+CD45+CX3CR37+ microglia. (C) Expression levels of indicated surface protein on CD11b+CD45+CX3CR37+ microglia in cerebral cortex (CTX) and hippocampus (HCP). (D, E) z-score standardized gene expression (normalized to reference gene) of indicated genes in cortex and hippocampus. (F) Total locomotion duration of Gata3fl/fl and Nmur1iCre-eGFP/+ Gata3fl/fl mice. (G) Open-field test (10 min) results, showing time spend in the center or near walls of Gata3fl/fl and Nmur1iCre-eGFP/+ Gata3fl/fl mice. (H) Total time of immobility during tail suspension test (6 min). (I) Duration of proximity (≥ 10 cm) during social interaction test (5 min). (J) Total number of visits in all corners and nose pokes by sex. The plots (A, B) show concatenated samples (n=4 mice), and (C) data are presented as Z-scores of respective MFI. The plots (A, B) show concatenated samples (A, B: n=4 mice). Analyses were performed at postnatal day 15 (D, E), 30 (D, E) or 8-18 weeks of age (A-J) by (A, I-females) Welch’s t test, (F, H-males) unpaired t test, (G) multiple unpaired t tests with Welch correction (False Discovery Rate Q=1.00%, two-stage step-up method of Benjamini, Krieger, and Yekutieli), (H-females, I-males) Mann-Whitney test. [file 12974_2025_3485_MOESM3_ESM.png]
